# Entwicklung der Katheterablation supraventrikulärer Tachykardien unter besonderer Berücksichtigung der Beiträge deutscher Ingenieure und Elektrophysiologen

**DOI:** 10.1007/s00399-024-01009-x

**Published:** 2024-02-28

**Authors:** Gerhard Hindricks, Verena Tscholl, Nikolaos Dagres, Philipp Attanasio, Martin Huemer

**Affiliations:** Deutsches Herzzentrum der Charité, Campus Charité Mitte, Berlin, DE Deutschland

**Keywords:** Katheterablation, Herzrhythmusstörungen, Supraventrikuläre Arrhythmien, Vorhofflimmern, Catheter ablation, Cardiac arrhythmias, Supraventricular arrhythmias, Atrial fibrillation

## Abstract

Die Entwicklung und klinische Implementierung der Katheterablation supraventrikulärer Tachykardien ist eine der herausragenden Errungenschaften moderner kardiovaskulärer Behandlung. Innerhalb von weniger als 40 Jahren ist es gelungen, für fast alle Formen von Vorhofrhythmusstörungen eine kurative und sichere Behandlungsstrategie zu entwickeln und flächendeckend zu implementieren. Deutsche Elektrophysiologen und Ingenieure haben einen wesentlichen Beitrag zu dieser wirklich herausragenden Erfolgsgeschichte in der Medizin geleistet. Diese Beiträge sollen im Kontext der zeitlichen Entwicklung dargestellt und gewürdigt werden. Ohne diese Beiträge wäre die Entwicklung der Ablationstechnologie und ihre weltweite Verbreitung nicht möglich gewesen. Sowohl die technologischen Beiträge wie auch die medizinisch-elektrophysiologischen Beiträge lagen in der absoluten Frontlinie der weltweiten Entwicklungen und haben einen wesentlichen Beitrag dazu geleistet, dass heute in jedem Jahr mehr als 500.000 Patienten mit symptomatischen und/oder bedrohlichen Herzrhythmusstörungen mithilfe der Katheterablation erfolgreich behandelt werden können. Ihnen allen sei für ihre Beiträge herzlich gedankt.

## Abstract (en)

The development and clinical implementation of catheter ablation of supraventricular tachycardia is one of the outstanding achievements of modern cardiovascular treatment. Over a period of less than 40 years, a curative and safe treatment strategy for almost all forms of atrial arrhythmias has been developed and implemented. German electrophysiologists and engineers have made a significant contribution to this truly outstanding success story in modern medicine. Their contributions should be appropriately acknowledged because without them, the development of ablation technology and its worldwide dissemination would not have been possible. Both the technological contributions and the medical–electrophysiological contributions were at the absolute forefront of worldwide developments and have made a significant contribution to the fact that today more than 500,000 patients with symptomatic and/or threatening cardiac arrhythmias can be successfully treated every year by use of catheter ablation. We would like to thank them all for their achievements.

Die Entwicklung der Katheterablation von Herzrhythmusstörungen gehört zu den spannendsten und auch atemberaubendsten Entwicklungsgeschichten in der kardiovaskulären Medizin. Über viele Jahrhunderte war die Behandlung von Herzrhythmusstörungen eine Domäne der medikamentös-antiarrhythmischen Therapie. Erst durch die Entwicklung chirurgischer Verfahren zur Behandlung von Herzrhythmusstörungen ergaben sich Möglichkeiten zur kurativen Therapie. Diese Entwicklungen waren ebenfalls spektakulär und werden in einem gesonderten Kapitel gewürdigt und behandelt.

Die Geschichte der Entwicklung der Katheterablation wurde durch einen im wahrsten Sinne des Wortes elektrischen Unfall eingeleitet, der sich im Rahmen einer diagnostischen invasiven elektrophysiologischen Untersuchung ergab: In dem Katheterlabor des französischen Kardiologen Guy Fontaine wurde im Rahmen einer wegen Kammerflimmerns notwendigen Defibrillation durch den versehentlichen Kontakt einer im Herzen liegenden Katheterelektrode mit einer Defibrillationselektrode durch die Schockabgabe ein kompletter AV-Block induziert. Diese Kasuistik wurde 1979 von Vedel und Fontaine als Komplikation einer elektrophysiologischen Untersuchung publiziert und von den amerikanischen Elektrophysiologen Melvin Scheinmann und John Gallagher aufgegriffen [1]. Gonzales und Scheinmann publizierten 1981 erste tierexperimentelle Daten zur therapeutischen Anwendung der Katheterablation zur Durchtrennung der atrioventrikulären Überleitung [2–4]. Bereits im Jahr darauf wurden erste klinische Ergebnisse zur Anwendung der DC-Gleichstromablation nahezu zeitgleich von Scheinmann und Gallagher vorgestellt. In den folgenden Jahren wurden von mehreren europäischen und nordamerikanischen Arbeitsgruppen Ergebnisse zur Gleichstrom-Katheterablation supraventrikulärer und atrioventrikulärer Tachykardien vorgestellt [5].

Neben Berichten zur durchaus erfolgreichen Anwendung der Methode mehrten sich aber auch Mitteilungen zu schweren Komplikationen, insbesondere Myokardrupturen und Perikardtamponaden, die auch zu Todesfällen führten [6].

Daraus entwickelte sich eine intensive Suche nach alternativen Energieformen zur Katheterablation, um eine sicherere Anwendung möglich zu machen. 1985 wurde von dem deutschen Ingenieur Dr. Peter Osypka der erste Hochfrequenzstromgenerator zur Ablation von Herzrhythmusstörungen entwickelt und vorgestellt. Die Technologie wurde klinisch in Deutschland erstmalig 1986 von der Arbeitsgruppe um Günter Breithardt aus der Universitätsklinik Düsseldorf erfolgreich eingesetzt [7–9]. Die Hochfrequenzstromablation erwies sich als deutlich sicherere Behandlungstechnologie im Vergleich zur DC-Gleichstromapplikation. Wesentliche Beiträge zur klinischen Entwicklung der HF-Katheterablation wurden von den deutschen elektrophysiologischen Arbeitsgruppen um Gerhard Steinbeck aus München sowie insbesondere von der Hamburger Arbeitsgruppe um Karl Heinz Kuck geleistet [10–13]. Parallel hierzu wurden von deutschen Arbeitsgruppen und auch nordamerikanischen Kollegen grundlegende biophysikalische Studien zu den Gewebewirkungen der Hochfrequenzstrom Ablation vorgestellt [14]. Mit zunehmend besserem Verständnis zum Umgang mit der Energiequelle weitete sich deren Einsatz bei unterschiedlichen Formen tachykarder Herzrhythmusstörungen aus. Borggrefe und Kollegen berichteten von der ersten erfolgreichen Durchtrennung einer rechtsgelegenen akzessorischen Leitungsbahn mittels Hochfrequenzstrom-Ablation, Kuck und Kollegen von der erfolgreichen Anwendung bei linksgelegenen akzessorischen Leitungsbahnen ([9, 13] siehe hierzu auch Beitrag von Borggrefe et al.). Insbesondere in den 1990er-Jahren setzte sich die Katheterablation mittels Hochfrequenzstrom bei unterschiedlichen Formen tachykarder Herzrhythmusstörungen in tatsächlich atemberaubender Art und Weise immer mehr durch [15].

Nach der erfolgreichen Anwendung zur Durchtrennung der atrioventrikulären Überleitung und akzessorischen Leitungsbahn konnte die Behandlungstechnik auch erfolgreich zur Modulation des AV-Knotens bei AV-Knoten-Reentry-Tachykardien, bei fokalen arteriellen Tachykardien und bei Vorhofflattern bzw. atrialen Reentry-Tachykardien eingesetzt werden (Abb. 1). Der insgesamt bemerkenswerteste und vielleicht auch wichtigste Durchbruch gelang aber sicherlich durch die Anwendung der Hochfrequenzstrom-Katheterablation zur Behandlung von Vorhofflimmern.

**Figure 1:**
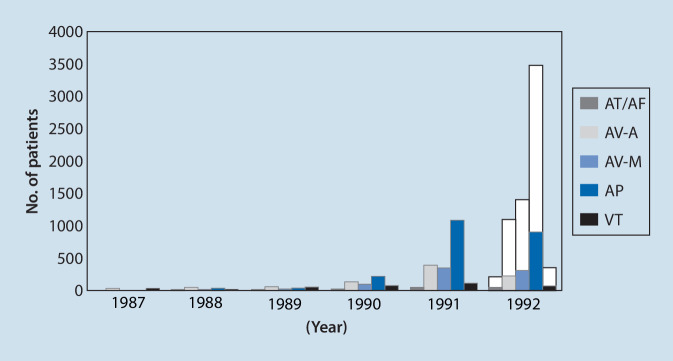

**Figure 2:**
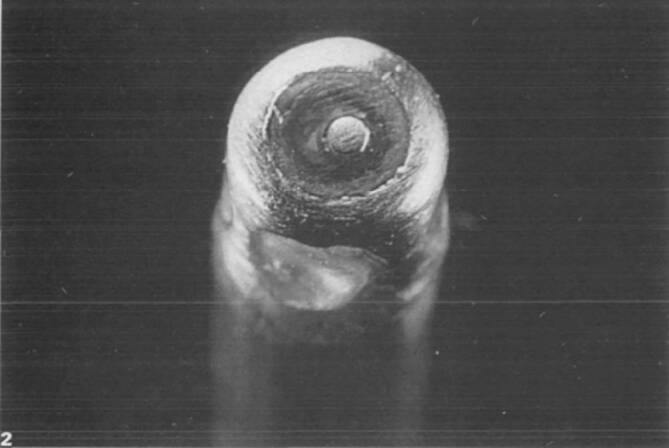

Die wesentlichen Schritte dieser Erfolgsgeschichte in Bezug auf die einzelnen Formen der Herzrhythmusstörungen werden im Folgenden nachgezeichnet. Außerdem erlaubt sich der Autor einige persönliche Bemerkungen und Mitteilungen zu interessanten Erfahrungen und Begegnungen auf dieser Entwicklungsstrecke.

## Technische, apparative und methodische Entwicklungen

Wie in der Einleitung bereits kurz dargestellt, wurde zur Katheterablation von Herzrhythmusstörungen zunächst die Gleichstromablation (auch DC-Ablation) eingesetzt. Hierzu wurde die im Bereich des vermuteten Zielgewebes im Herzen platzierte Katheterelektrode über einen Adapter mit der Elektrode eines handelsüblichen Defibrillators verbunden. Als Gegenelektrode wurde eine großflächige Hautelektrode am Patienten fixiert und die Energieabgabe unipolar durchgeführt. Im Moment der Stromabgabe wurden innerhalb von Millisekunden Spannungen von mehreren 1000 V über die Katheterelektrode dem Myokard zugeführt. Durch die Energieabgabe entstehen im Bereich der Katheterelektrode erhebliche Druckwellen, die zu sogenannten barotraumatischen Gewebeeffekten führen. Einerseits konnte so zwar das Zielgewebe erfolgreich zerstört werden, als Folge des Barotraumas stellten sich aber auch gefährliche Komplikationen wie Myokardperforationen und Perikardtamponaden ein. Die relativ hohe Komplikationsrate der Eingriffe war somit eine erhebliche Limitierung der Nutzbarkeit.

**Figure 3:**
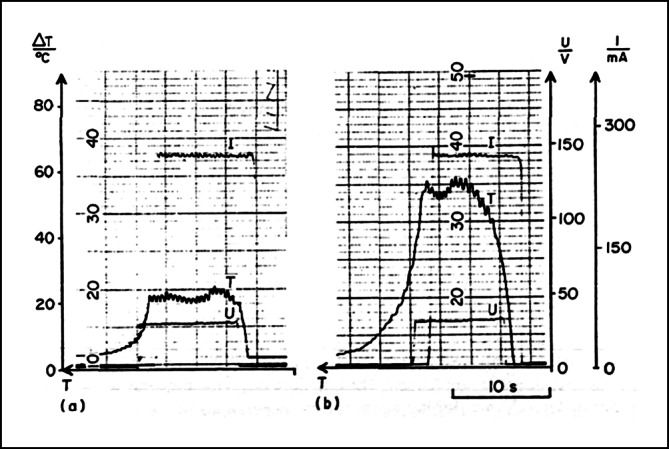

Als alternative Energiequelle wurde 1985 von dem Ingenieur Dr. Peter Osypka die Hochfrequenzstromenergie zur Katheterablation mit der Vorstellung des Hochfrequenzstrom-Generators HAT 100 eingeführt. Mit dem Generator konnte sinusförmiger nichtmodulierter Hochfrequenzstrom in 10 Leistungsstufen abgegeben werden. Die Energieabgabe erfolgte analog zur Gleichstromablation in unipolarer Elektrodenkonfiguration über die endständige Elektrode eines handelsüblichen diagnostischen Elektrodenkatheters. Durch die Stromabgabe wurde im Übergangsbereich zwischen der Katheterelektrode und dem Herzmuskelgewebe Widerstandswärme erzeugt, die zur Ausbildung einer Koagulationsnekrose in der Umgebung des Elektrodenkatheters führte. Die Stromabgabe war mit dem einfachen Generator schwierig zu kontrollieren, und es stellten sich immer wieder Überhitzungen der Katheterelektrode ein, die teilweise zu *Verkohlungen* (Karbonisation) mit zum Teil erheblichen Isolationsdefekten des Elektrodenkatheters führten. Um die Überhitzung der Katheterelektrode zu verhindern, wurden spezielle Elektrodenkatheter entwickelt, die mit Thermistoren in der Katheterspitze ausgestattet waren, um die Stromabgabe bei zu starker Temperaturentwicklung zu limitieren (Abb. 2). Die erste erfolgreiche klinische Anwendung der temperaturkontrollierten Ablation wurde 1988 von der Arbeitsgruppe in Münster durchgeführt. Die sogenannte temperaturgesteuerte Hochfrequenzstromablation erlaubte eine effektivere und sichere Applikationsbehandlung und setzte sich als Standard der Hochfrequenzstromablation in den nächsten Jahren durch ([14, 16, 17]; Abb. 3 und 4).

**Figure 4:**
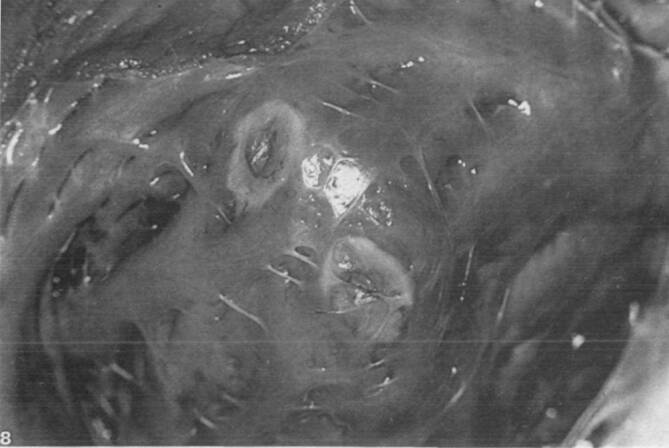

Alternativ wurden auch andere Energiequellen wie Laserenergie und später auch fokussierter Ultraschall hinsichtlich der Möglichkeiten zur Ablationsbehandlung untersucht. Letztlich konnte sich keine der alternativen Energiequellen durchsetzen, und die Hochfrequenzstrom-Katheterablation setzte den technologischen Standard für die Behandlung von Herzrhythmusstörungen. Die in Deutschland entwickelte Technologie und auch die ersten klinischen Anwendungen wurde insbesondere von den Arbeitsgruppen um Borggrefe und Breithardt so wie Kuck und Mitarbeiter befördert [16–19]. Wesentliche Beiträge zur technologischen Verbesserung der Applikationsmethode wurden aber auch von den amerikanischen Elektrophysiologen David Haines und Hiroshi Nakagawa geleistet [20, 21].

Die Entwicklung der Katheterablation wäre jedoch nicht möglich gewesen ohne die sich parallel entwickelnde Verfügbarkeit von sogenannten elektroanatomischen Mappingsystemen, die eine Verbindung zwischen Elektrophysiologie und Anatomie in bis dahin nicht vorstellbarer Genauigkeit möglich machten. Der wesentlichste Beitrag in diesem Bereich ist sicherlich Shlomo Ben-Haim durch die Entwicklung des Carto-Systems zuzuschreiben. Neben der hohen Präzision dieses ersten nichtfluoroskopischen Mappingsystems konnte so die Strahlenbelastung für die behandelten Patienten und auch für die Behandler substanziell reduziert werden [22].

## Katheterablation der atrioventrikulären Überleitung

Nach den oben bereits beschriebenen ersten experimentellen und klinischen Untersuchungen zur Gleichstromablation durch Scheinman und Gallagher wurde die erste Anwendung in Deutschland 1983 durch Lüderitz und Manz beschrieben [5]. Die erste erfolgreiche Anwendung der Hochfrequenzstrom-Ablation zur Durchtrennung der atrioventrikulären Überleitung erfolgte 1985 von Budde und Mitarbeitern [8].

## Kathetermodulation des AV-Knotens bei AV-Knoten-Reentry-Tachykardien

Erste erfolgreiche Anwendungen der Katheterablation zur Modulation des AV-Knotens bei AVNRT wurden 1998 von Haissaguerre und Mitarbeitern vorgestellt. Die Untersuchungen wurden zunächst durch Gleichstromablation der retrograden Leitung des AV-Knotens durchgeführt. Die Methode erwies sich als durchaus effektiv zur kurativen Behandlung der Tachykardien, ging aber mit einer relativ hohen Häufigkeit kompletter AV-Blockierung und nachfolgender Schrittmacherpflichtigkeit einher [23]. 1992 stellten Jackman und Mitarbeiter die Methode der AV-Knotenmodulation durch selektive Ablation der langsam leitenden Anteile des AV-Knotens vor. Die Methode hatte den wesentlichen Vorteil dadurch, dass die Häufigkeit höhergradiger AV-Blockierungen als Folge der Behandlung im Vergleich zur Ablation der schnell leitenden Anteile des AV-Knotens erheblich geringer war [24]. Diese Methode hat sich dann als Standard für die Modulation des AV-Knotens erfolgreich durchgesetzt. Mittlerweile konnten viele 10.000 Patienten durch diesen sicheren und kurativen Eingriff von ihren hochsymptomatischen Herzrhythmusstörungen geheilt werden. Die Modulation des AV-Knotens kann heute in geübter Hand in nahezu allen Fällen erfolgreich und sicher durchgeführt werden.

## Katheterablation von typischem Vorhofflattern und atrialen Reentry-Tachykardien

Die Entwicklung der Katheterablation zur erfolgreichen Behandlung von rechtsatrialem, sog. typischem Vorhofflattern ist ohne die pathophysiologischen Untersuchungen von Waldo und Mitarbeitern nicht denkbar [25]. Diese Arbeitsgruppe legte grundlegende Ergebnisse zur Beschreibung des Reentry-Kreises bei typischem Vorhofflattern vor und schuf damit die Grundlage für die Entwicklung einer Strategie zur erfolgreichen Behandlung mittels Katheterablation. Diese wurde von Saoudi und Mitarbeitern aufgegriffen und in ein Ablationskonzept durch die Applikation linearer Läsionen zwischen dem inferioren Bereich des Trikuspidalklappenanulus und der Mündung der Vena cava inferior entwickelt [26]. Saudi und Mitarbeiter konnten zeigen, dass durch eine Läsion entlang dem rechtsatrialen Isthmus der Reentry-Kreis erfolgreich unterbrochen und das Wiederauftreten von typischem Vorhofflattern effektiv verhindert werden konnte. Vergleichbare Behandlungskonzepte wurden später dann auch für die erfolgreiche Behandlung anderer rechts- und linksatrialer Reentry-Tachykardien entwickelt und etabliert.

## Katheterablation von Vorhofflimmern

Mit der Entwicklung einer chirurgischen Technik zur Behandlung von Vorhofflimmern leitete der Herzchirurg Jimmy Cox eine neue Ära in der Behandlung von Vorhofflimmern ein. Cox entwickelte nach umfangreichen experimentellen Vorarbeiten die sog. Maze-Prozedur. Im Rahmen der Operation wurden der rechte und der linke Vorhof durch multiple Inzisionen in isolierte Kompartimente unterteilt und dadurch das Auftreten von Vorhofflimmern verhindert. Teil der Maze-Prozedur war auch die komplette Isolation der Pulmonalvenen [27]. Der amerikanische Elektrophysiologie John Schwarz war der Erste, der in klinischen Studien versuchte, das Therapiekonzept der chirurgischen Maze-Operation in eine Katheterprozedur zu übertragen. Die Ergebnisse dieser Studien wurden 1986 im Rahmen der Jahrestagung der Amerikanischen Gesellschaft für Kardiologie vorgestellt, aber leider nie als volles Manuskript publiziert. Inspiriert durch die Untersuchungen und Befunde von John Schwarz arbeitete der französische Elektrophysiologe Michelle Haissaguerre an Ablationskonzepten für die Therapie von Vorhofflimmern [28]. In einer ersten Mitteilung wurde die Beobachtung von sog. fokalem rechtsatrialem Vorhofflimmern und einer erfolgreichen Ablation bei 3 Patienten beschrieben [28]. Der Durchbruch sowohl im pathophysiologischen Verständnis des Vorhofflimmerns wie auch hinsichtlich der Entwicklung von Therapiestrategien gelang Haissaguerre und Mitarbeitern durch die Identifizierung von Pulmonalvenenfoci und deren Bedeutung für das Auftreten von Vorhofflimmern. In einer wirklichen Meilenstein-Arbeit publizierte Haissaguerre diese Befunde 1993 im *New England Journal of Medicine* [29]. Damit war die Entwicklung des Therapiekonzeptes der Pulmonalvenenisolation zur Behandlung von Vorhofflimmern vorgestellt. Parallel hierzu wurden Elektrodenkatheter entwickelt, durch welche die Elektrophysiologie der Pulmonalvenen abgebildet werden konnte (sog. Lasso-Katheter). Herausragende Beiträge zum besseren Verständnis der Elektrophysiologie der Pulmonalvenen und der Entwicklung effektiver Ablationsstrategien wurden von der Arbeitsgruppe um Karl Heinz Kuck aus Hamburg geleistet. Carlo Pappone stellte als Erster das Konzept der Pulmonalvenenisolation durch zirkumferenzielle Ablation auf Vorhofebene vor [29]. Dieses Konzept wurde durch die Nutzung elektroanatomischer Mappingtechnologie zur klinischen Anwendungsreife geführt. Das Prinzip der kompletten Isolation der Pulmonalvenen hat sich als effektives Therapiekonzept für die Katheterablation von Vorhofflimmern mittlerweile gut etabliert. Von unterschiedlichen Arbeitsgruppen wurden alternative Ablationskonzepte wie die gezielte Ablation fraktionierter arterieller Potenziale oder auch durch Ablation sog. Rotoren klinisch untersucht. Beide Methoden konnten sich aufgrund geringer Effektivität nicht durchsetzen. Auch die Ergänzung der Pulmonalvenenisolation durch sog. lineare Läsionen, beispielsweise entlang des Mitralklappenanulus oder als sog. linksatriale Dachlinie, konnte sich nicht durchsetzen. Die einzige Technik, die in einigen Studien Vorteile gegenüber der alleinigen Pulmonalvenenisolation zeigen konnte, war die sog. Substratmodifikation durch gezielte Ablation atrialer Myokardbezirke, die sich durch sog. Low-Voltage-Areale auszeichnen. Das Konzept der atrialen Kardiomyopathie wurde u. a. von Hans Kottkamp entwickelt und klinisch etabliert [30]. Piorkowski und Mitarbeiter konnten im Rahmen einer randomisierten Studie zeigen, dass die gezielte Ablation dieser Areale zu einer signifikanten Verbesserung der Ablationsergebnisse führt [31].

Die Entwicklung der Kryoablation in den Jahren 2000 bis 2010 wurde wesentlich von der deutschen Elektrophysiologie mitbestimmt. Erste größere Untersuchungsserien wurden von Ellen Hoffmann und ihrer Arbeitsgruppe aus München vorgestellt [32]. Die erste große randomisierte Studie, die eine Gleichwertigkeit der Kryoablation zur Katheterablation mit Hochfrequenzstrom zeigte, wurde von Karl-Heinz Kuck geleitet und durchgeführt: Fire and Ice [33].

Die wesentliche aktuelle technologische Entwicklung im Bereich der Katheterablation von Vorhofflimmern ist sicherlich der Einsatz der sog. „pulsed-field ablation“ zur linksatrialen Ablation [34, 35]. Im Gegensatz zur Katheterablation mit Hochfrequenzstrom oder zur Kryoablation wird die Läsionsinduktion bei Einsatz der PFA-Energie fast ausschließlich durch eine direkte elektrische Schädigung der Zellmembranen ohne wesentliche thermische Effekte induziert. Vorteil der PFA-Technologie ist darüber hinaus, dass eine Schädigung des Ösophagus, der Pulmonalvenen (Pulmonalvenenstenose) oder auch des Nervus phrenicus im Vergleich zu den thermischen Ablationsmethoden deutlich niedriger zu sein scheint. Gleichzeitig kann die Ablationsbehandlung schneller durchgeführt werden. Im Moment laufen erste größere klinische Studien, die vergleichend die Wirksamkeit thermischer und elektrischer Ablationsmethoden untersuchen. Außerdem wurden in den letzten Jahren Studien zur Ablationsbehandlung komplexer Herzrhythmusstörungen durch gezielte Strahlenapplikation mittels Linearbeschleunigern, die ansonsten in der Krebsbehandlung eingesetzt werden, durchgeführt: die transkutane Ablation von Herzrhythmusstörungen [36, 37]. Es geht also immer weiter mit den technischen und technologischen Entwicklungen in der Rhythmologie. Wir sind gespannt!
